# In vitro and in vivo assessment of the anti-inflammatory activity of olive leaf extract in rats

**DOI:** 10.1007/s10787-023-01208-x

**Published:** 2023-04-08

**Authors:** Nada Fayez, Waleed Khalil, Essam Abdel-Sattar, Abdel-Fattah Mohamed Abdel-Fattah

**Affiliations:** 1 Pharmacology Department, Faculty of Veterinary Medicine, Suez Canal University, Ismailia, 41522 Egypt; 2 Pharmacognosy Department, Faculty of Pharmacy, Cairo University, Kasr el Aini St., Cairo, 11562 Egypt

**Keywords:** Olive leaves, Carrageenan, Diclofenac, Anti-inflammatory, Paw edema

## Abstract

Inflammation is a complex and crucial process that protects the body against pathogens. Here in our study, we propose to scientifically justify the anti-inflammatory activity of olive leaf (OL). Initially, we ensured the safety of olive leaf extract (OLE) through acute oral administration of graded doses up to 4 g\kg in Wistar rats. Thus, the extract was considered generally safe. We also evaluated the ability of the extract to reduce carrageenan-induced rat paw edema. Compared to diclofenac sodium (10 mg/kg PO), OLE showed significant (*P* < 0.05) anti-inflammatory activity, showing the maximum inhibition percentage at the fifth hour of measurement at 42.31% and 46.99%, at doses of 200 and 400 m/kg, respectively, compared to 63.81% for the standard drug. To elucidate the potential mechanism, we measured TNF, IL-1, COX-2 and NO inside the paw tissue. Interestingly, OLE at all tested doses reduced the concentration of TNF and IL-1 to a level that was lower than that obtained by the standard drug. Additionally, OLE at the dose of 400 mg/kg reduced the levels of COX-2 and NO inside the paw tissue to a level that was statistically equivalent to the level observed in the normal control group. Finally, olive leaf extract at doses of 100, 200 and 400 mg/kg doses significantly (*P* < 0.05) inhibited the heat-induced hemolysis of RBCs membrane by 25.62, 57.40 and 73.88%, respectively, compared to 83.89% produced by aspirin. Consequently, we concluded that olive leaf extract has a significant anti-inflammatory activity through the reduction of TNF, IL-1, COX-2 and NO.

## Introduction

Inflammation is a complex process mediated by the body to defend against inflammogens such as infection, or any tissue injury caused by physical, chemical or biological stimuli. While it seems, a normal physiological process, when homeostatic control is lost, inflammation is turned into a damaging tool that contributes to the worsening of major diseases and the appearance of serious pathological complications. Hopefully, there are many emerging synthetic compounds which treat inflammation and several related diseases (Braz-Silva et al. 2019; Nathan and Ding 2010).

Although synthetic drugs can treat several diseases, they also cause severe adverse effects on the human and animal body. These adverse effects of chemical drugs are well documented, ranging from allergy, and teratogenesis to long-term genetic damage that increases the susceptibility of persons to the development of cancer (Popiolek and Porebski 2022; Rannug et al. 1995). In the United States of America, about 8% of hospital admissions are due to side or adverse effects of manufactured drugs (George 2011). Each year also thousands of people die from apparently “safe” over-the-counter drugs, (Karimi et al. 2015). On the other hand, deaths or hospitalizations due to herbs are so rare that they are hard to find. Even, the Poison Control Centers (PCCs) of the United States do not include side or adverse reactions to herbs in their database (Nasri 2013). Therefore, people each year turn to plant remedies because they believe herbal medicine is free from undesirable side effects (Haq 2004; Kazemipoor et al. 2012; Nasri 2013). And there has been a renewed interest in natural product research over the past ten years. (Lahlou 2013). According to the reports of WHO, 2022, around 40% of currently used pharmaceutical products originated from natural substances, highlighting the vital importance of conserving biodiversity and sustainability. So, many biowaste-related issues like nutritional, economical and environmental problems can be solved through the bioconversion of these wastes (Sagar et al. 2018). In addition, the exploitation of plant wastes/by-products for the recovery of added-value compounds opens new opportunities for industrial development and waste management.

The olive plant, Olea europaea L. belongs to the Oleaceae or the dicotyledons family. The olive tree is one of the most important plants in oil production all over the world and it has great historical and commercial importance, especially in Mediterranean countries (Vossen 2007; Zhang et al. 2018). It is widely cultivated in Egypt, particularly, in Sinai. Many studies reported that Olive leaf is used in folk medicine to cure many diseases such as diabetes, hypertension, gallstones and inflammation (Al-Khalil 1995; Ali-Shtayeh et al. 2012) and it is relatively safe as an antioxidant to treat glycemia and lipidemia (Acar-Tek and Ağagündüz 2020). Olive oil extracts have been extensively investigated in recent decades for both nutritious and medicinal interests (Ghanbari et al. 2012). On the other hand, the current use of olive leaves is very rare, and it is still considered a by-product of olive oil production.

To date, information about the effectiveness of olive leaf extract (OLE) especially those regarding its anti-inflammatory activity is exclusively derived from folk medicine as well as historical beliefs. Consequently, the current study was proposed to detect to what extent the extract is relatively safe and to elucidate the mechanism through which the anti-inflammatory activity is produced, in comparison with a standard drug.

## Materials

### Plant collection and identification

Fresh leaves of the olive plant were collected from the trees, cultivated at the experimental station of the agriculture research center (October 2020) and identified by Dr. Amr Salah, Associate Professor at Olive Research Department, Agriculture research center, Giza. Olive leaves*.* (Sp. # OE 10.10.2020) were deposited at the Department of Pharmacognosy, Faculty of Pharmacy, Cairo University, Egypt. The leaves were washed with water and dried under shade.

### Chemicals and drugs

Carrageenan λ (Sigma Lambda, USA) and diclofenac sodium (Declophen^®^) were obtained from Pharco Pharmaceutical (Alexandria, Egypt); ELISA kits (TNFα and IL-1β) were purchased from the ABclonal company (Immuno-Biological Laboratories (IBL), catalogue number: RK00029).

Rat Cyclooxygenase 2 (COX-2) ELISA Kit (catalogue number: MBS020734) and Nitric Oxide Microplate Assay Kit (catalogue number: MBS8243214) were purchased from MYBIOSOURCE.

### Animals and ethical approval

Wistar albino rats (male and female) weighing 150–200 g were purchased from the lab animal house College of Pharmacy-Suez Canal University. Rats were adapted for one week at the laboratory conditions of the Pharmacology Department, College of Veterinary Medicine, Suez Canal University to minimize animal stress. Rats were housed in standard translucent cages (4 animals/cage) under controlled standard conditions (23 ± 3 °C, 60 ± 10% relative humidity, 12 h light/dark cycle) with free access to a standard rodent pellet diet and tap water. After acclimation for 1 week, healthy animals were randomly assigned into 4 groups (6 rats per group). They were provided with free access to a standard rodent pellet diet as well as free tap water. Animal handling and care were conducted according to the National Research guidelines (Health, 1985). Prior to performing any experimental procedures, our pharmacological protocols were ethically approved by the institutional ethical committee, Faculty of Veterinary Medicine, Suez Canal University with approval No (202,014).

## Methods

### Plant preparation and standardization

Using boiling water and Ultra-Turrax T50 high-speed homogenizer (IKA, Staufen, Germany), an olive leaves sample (500 g) was extracted (2 × 2000 ml), followed by solvent evaporation under reduced pressure, and the dried extract was kept at 4 °C.

Olive leaves extract (50 mg) was dissolved in methanol and completed the volume to 25 mL, followed by injection into HPLC (in triplicate). 

### ***Chromatographic conditions***

Using Agilent 1260 Infinity Quaternary LC, equipped with Agilent 1260 Infinity; Quaternary Pump (G1311B), and Diode Array Detector (G1315D, VL) coupled to Agilent Open LAB Chem Station B.04.03 software, chromatographic analysis was carried out. Analytes were separated on Agilent Zorbax Eclipse XDB-C18 (250 mm × 4.6 mm i.d., 5_mparticle diameter) protected with Agilent Zorbax XDB-C18 pre-column (Agilent Technologies, Palo Alto, CA, USA). The flow rate of 1.0 mL/min and the UV detector was set at 280 nm. Separation was carried out with acetonitrile (A) and 0.1% trifluoroacetic acid in water (B) and gradient elution program at a flow rate of 1 ml/min starting with 20% A in B for 5 min, 20–30% A/B in 5 min, 30–50% A in B in 5 min, 50–100% A in B in 10 min followed by washing with 100% A for 5 min.

### Establishment of the standard calibration curve

**Fig. 1:**
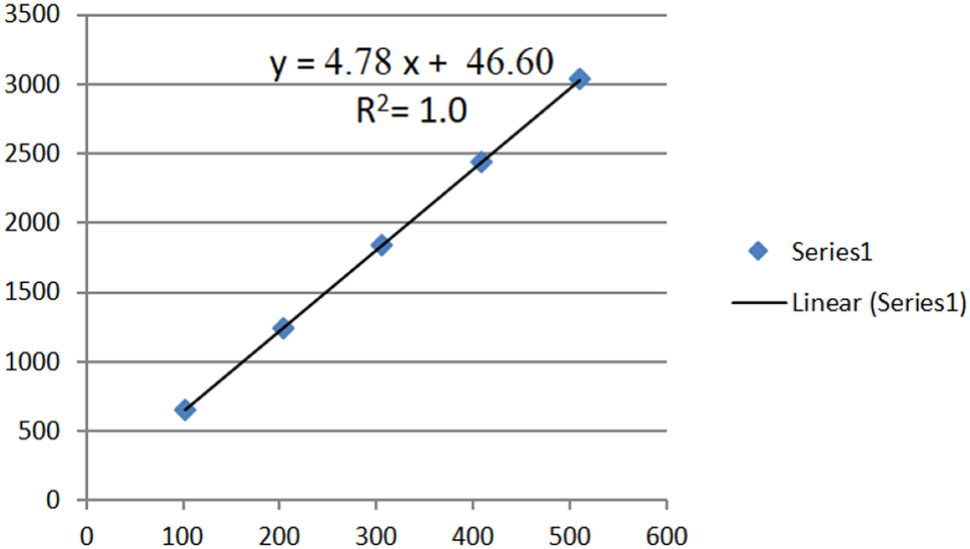
Standard calibration curve of oleuropein

A standard calibration curve of standard oleuropein **(**Fig. 1**)** was established in a concentration ranging from 100.0 to 500 µg/ml.

### Acute oral toxicity

Acute oral toxicity was performed in accordance with the method of the Organization for Economic Co-operation and Development (OECD), (Co-operation and Development 2008). Five male and five female rats were randomly assigned to the experimental and control group, after fasting overnight with free access to water. The aqueous extract of OLE was administered orally using rat oral gavage in different gradual doses (up to 4000 mg/kg BW). For successive four days, the animals were regularly monitored for any alterations in their autonomic or behavioral responses. Once a day, a clinical observation was carried out to look for any signs of death, illness, or treatment-related side effects, such as changes in behavior, salivation, diarrhea, drowsiness, or coma. The animals were maintained under observation for 14 days to record any deaths or harmful effects that might have been delayed. (Walum 1998).

## Anti-inflammatory activities

### In vivo anti-inflammatory activity

#### Carrageenan-induced paw edema

The anti-inflammatory activity of OLE was measured using the carrageenan-induced paw edema method in rats according to Jia et al. (2005). 0.1 ml of 1% freshly prepared suspension of carrageenan was injected sub-planter into the right footpad of the rats’ hind paw in all groups to induce paw inflammation and edema. The left hind paw was not treated, and it was taken as a control to calculate the change in paw thickness. Carrageenan caused visible redness and pronounced swelling that was well developed by 3 h and persisted till the end of the experiment. The progression of paw edema was measured at 0, 1, 2, 3, 4, and 5 h intervals using Vernier digital caliper. The previously adapted animals were divided into five groups each (*n* = 6). One hour prior to carrageenan injection, the animals were pretreated with OLE at doses (100, 200 and 400 mg/kg) to the first three groups, respectively. The rest two groups received distilled water as a vehicle (control 10 ml/kg) and diclofenac (10 mg/kg) as reference drugs, respectively.

Compared to the control groups, received the vehicle, the percent of the edema’s inhibition was calculated as per the formula shown below:

$${\text{Inhibition }}\% \, = \frac{{{\text{Change in control}} - {\text{ change in treatment }} \times 100}}{{\text{Change in control}}},$$

where the change of paw thickness values was calculated from the difference between the left and the right paw volumes.

#### Assessment of the IL-1β, TNF-α, NO and COX-2 levels in the rat paw.

TNF-α, IL-1β, NO and COX-2 levels in the paw tissues were evaluated by enzyme-linked immunosorbent assay (ELISA) as described previously by Nacife et al. (2004). Five hours after the induction of inflammation, the collected samples were weighed; stored at − 20 °C to be processed for TNF-α, IL-1β, NO and COX-2 levels in the paw tissues were evaluated by enzyme-linked determinations. The subcutaneous tissue of the right hind paw and that surrounding the tarso-tibial joints were homogenized in phosphate buffer saline (PBS) at pH = 7.4. The homogenates were centrifuged at 10,000 rpm at 4 °C for 10 min, and then ELISA was used to measure the levels of TNF-α, IL-1β, NO and COX-2 in the supernatants, according to the manufacturer’s instructions. Briefly, the microtiter plate provided in the ELISA kits has been pre-coated with a monoclonal antibody specific for each cytokine. The homogenate was carefully pipetted into the wells. Then, all unbound material was removed after washing. The immobilized monoclonal antibody in the plate-bound target cytokine in standards and samples and a secondary biotinylated polyclonal antibody were added. This antibody is recognized by a streptavidin-antibody, which is recognized by a streptavidin-peroxidase substrate. Finally, the color development was stopped, and an ELISA reader was used to read the plates at 490 nm. Standard curves were established, and the concentration of each parameter was calculated using the standard curves.

### In vitro experiments

#### Red blood cell suspension (RBCs)

To prepare RBCs suspension, blood was obtained from apparently healthy anaesthetised rats and collected in heparinized tubes. Using the centrifuge (3000 rpm at room temperature for 15 min), the plasma (supernatant) was clearly removed and red blood cells (RBCs) were washed three times using PBS (pH 7.4). Using PBS, the 10% (v/v) cell suspension was prepared and used in the assays.

#### Heat-induced hemolysis

For the control tubes, only saline solution was used. The reaction mixture (2 mL) contains 1 mL of 10% RBC suspension and 1 mL of test substances in various concentrations (standard drugs or plant extracts). After 30 min at 56 C, all tubes were fully cooled with tap water. After that, the test tubes were centrifuged for five minutes at 2500 rpm, and the supernatant absorbance was measured at 560 nm. (Sakat et al. 2010). The experiment was conducted in triplicate for all test specimens. The inhibition percent or acceleration of haemolysis in tests was calculated using the following formula:

$$\% {\text{ inhibition of hemolysis}}\, = \,100 - \, [1 - \left( {OD_{2} - OD_{1} / \, OD_{3} - OD_{1} } \right)],$$

whereOD_1_ equals the test sample unheated,OD_2_ equals the test sample heated and treated,OD_3_ equals control heated sample.

### Statistical analysis

All data were expressed as mean ± SE and statistically analyzed using Statistical Package for Social Science (SPSS) version 25.0. The statistical significance of differences among different study groups was evaluated using one-way analysis of variance (ANOVA), Differences were considered significant when the *p* value > 0.05. To determine differences between means of treatments at significance rates of 0.05, we used Duncan’s multiple range test.

## Results

### HPLC analysis of olive leaf extract

**Fig. 2:**
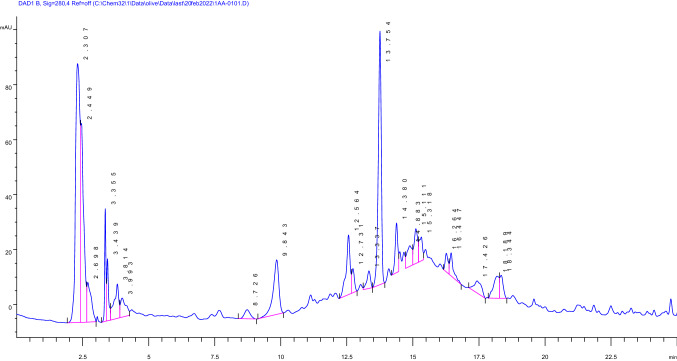
HPLC chromatographic separation of olive extract

From the standard calibration curve and the equation *Y* = 4.78*X* + 46.60, the concentration of oleuropein was determined to be 7% **(**Fig. 2**).**

### Acute oral toxicity of olive leaf extract

The Aqueous extract of OL did not cause any toxic symptoms or mortality at a single graded dose of up to (4000 mg/kg BW) in Wistar rats of both sexes. No observable behavioral changes occurred during the experiment, in which the animals were observed for successive 14 days. Furthermore, no drug-related morbidity or mortality occurred during the experiment period, and hence the drug was considered non-toxic and safe for further pharmacological screening.

## Anti-inflammatory activities of olive leaves extract:

### In vivo anti-inflammatory activity

#### Treatment with olive leaf extract significantly reduced the mean paw volume in rats

The anti-inflammatory potential of OL extract was evaluated using carrageenan-triggered inflammation in rats. The intra-plantar injection of carrageenan substantially increased the thickness of the rat paw. Olive leaf extract significantly (*P* < 0.05) reduced paw inflammation in a dose-dependent manner. OLE at doses 200 and 400 mg/kg showed the highest inhibition percentage at the fifth hour of measurement, reaching 42.31 and 46.99%, respectively, compared to 63.81% obtained by the standard drug. Notably, the aqueous extract at all tested doses exerted its anti-inflammatory action from the first hour of measurement Table 1.

**Table 1:** Changes and the inhibition percentage in rats paw thickness in carrageenan-induced inflammation

Groups	Dose (mg/kg)	Change in paw thickness mm (hours post treatment)				
		1st	2nd	3rd	4th	5th
Saline	–	2.28 ± 0.27^a^	2.54 ± 0.39^a^	3.59 ± 0.43^a^	3.22 ± 0.29^a^	3.11 ± 0.68^a^
Control positive diclofenac sodium	10	1.32 ± 0.26^bc^ (38.66)	1.29 ± 0.26^ cd^ (55.71)	1.31 ± 0.19^ef^ (61.78)	1.36 ± 0.23^d^ (60.51)	1.22 ± 0.28^c^ (63.81)
OLE	400	1.58 ± 0.17 ^bc^ (27.66)	1.90 ± 0.12^abc^ (25.38)	2.28 ± 0.12^ cd^ (32.46)	2.29 ± 0.06^bc^ (32.37)	1.78 ± 0.17^bc^ (46.99)
	200	1.60 ± 0.23^abc^ (16.71)	2.21 ± 0.42^ab^ (14.97)	2.69 ± 0.24^bc^ (19.99)	2.615 ± 0.13^bc^ (25.92)	2.045 ± 0.37^bc^ (42.31)
	100	2.00 ± 0.17^ab^ (4.58)	2.57 ± 0.42^a^ (1.90)	3.07 ± 0.24^ab^ (8.19)	2.88 ± 0.27^ab^ (16.20)	2.17 ± 0.23^ab^ (36.99)

Each value is the mean SEM for 6 mice. Different letters within the count column mean statistical significance at (*P* < 0.05)

The percentage of inhibition values are given in parentheses

#### Olive leaf extract significantly reduced the concentration of tumor necrosis factor-alpha (TNF-α) and interleukin 1-beta (IL-1) levels in the rat paw tissue

**Fig. 3:**
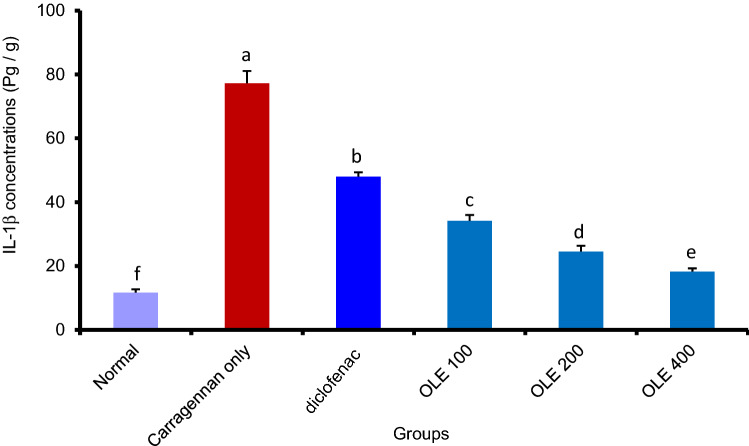
Olive leaf extract (OLE) significantly reduced IL-1 levels in carrageenan injected paw. Each value is the mean ± SEM for 6 rats per group. Above each column, different letters mean statistical significance at (*P* < 0.05)

**Fig. 4:**
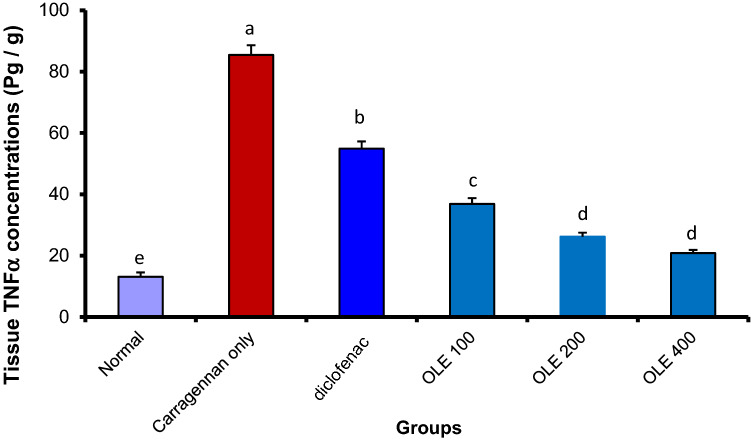
Olive leaf extract (OLE) decreased TNF levels significantly in carrageenan injected paw. Each value is the mean ± SEM for 6 rats per group. Above each column, different letters mean statistical significance at (*P* < 0.05)

The rat paw homogenate was used to quantify the amount of TNF-α and IL-1. While The sub-plantar injection of carrageenan increased the concentration of IL-1 and TNF-α inside the paw tissue, OLE (100–400 mg/kg) significantly (*p* > 0.05) reduced the tissue concentration of TNF-α (20.83 ± 1.1–36.88 ± 1.9) & IL-1 18.23 ± 1.04—34.18 ± 1.30) in a dose-dependent manner. Interestingly, the aqueous extract at all doses showed a significant reduction of TNF-α and IL-1 concentration more than the standard drug.** (**Figs. 3 and 4**)**.

#### Olive leaf extract substantially reduced cyclooxygenase 2 (COX-2) and nitrous oxide (NO) inside the inflamed paw tissue

**Fig. 5:**
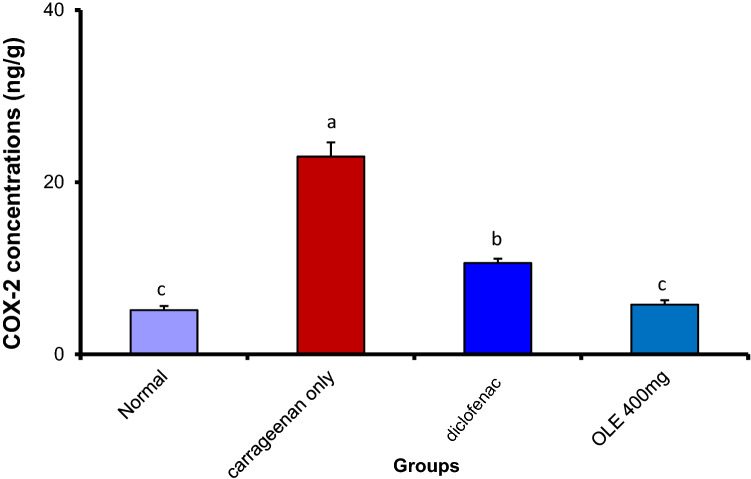
Olive leaf extract (OLE) reduced significantly COX-2 levels in carrageenan injected paw. Each value is the mean ± SEM for 6 rats per group. Above each column, different letters mean statistical significance at (*P* < 0.05)

**Fig. 6:**
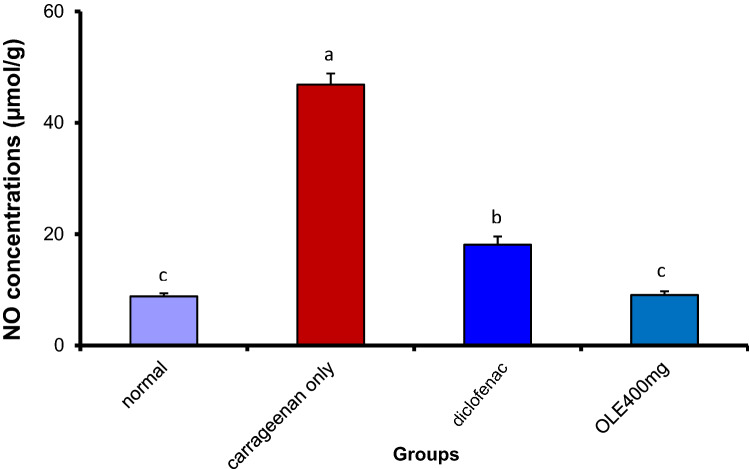
Olive leaf extract (OLE) decreased NO levels substantially in carrageenan injected paw. Each value is the mean ± SEM for 6 rats per group. Above each column, different letters mean statistical significance at (*P* < 0.05)

There was a clear significant increase of both COX-2 and NO in the carrageenan-treated group. Notably, OLE at the dose of 400 mg/kg induced a marked reduction in the level of COX-2 and NO that was statistically equivalent to the concentration of the normal group. The standard drug (Declophen) also reduces the concentration of both COX-2 and NO to reach 10.60 ± 0.51 and 18.09 ± 1.49, respectively. However, its effect was surpassed by the Aqueous extract of the plant. (Figs. 5, 6).

### *In vitro* anti-inflammatory of olive leaf extract

#### Olive leaf extract (OLE) protected the erythrocyte membrane against heat-induced haemolysis

**Fig. 7:**
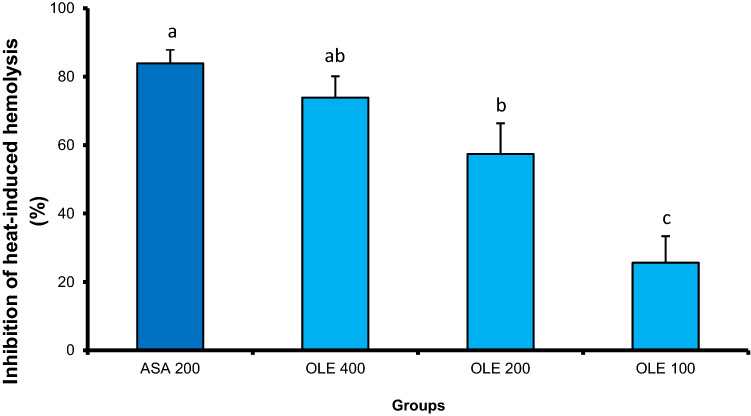
Olive leaf extract (OLE) inhibited significantly heat-induced haemolysis. Results are expressed as mean ± SE. Different superscripts, within the same column mean statistical significance at (*P* < 0.05)

Overall, the results of the erythrocyte membrane stabilization showed that OLE (100–400 g/kg, PO) showed a potent ability to protect the RBCs membrane against heat-induced haemolysis in a dose-dependent manner. Compared to the aspirin-treated group, there were no statistically significant differences between the percentage of inhibition produced by 400 g/kg of OLE and that produced by 200 g of aspirin., Olive leaf extract at doses of 100, 200 and 400 g/kg dose-dependently and significantly inhibited the heat-induced hemolysis of RBCs membrane by 25.62, 57.40 and 73.88%, respectively, compared to 83.89% produced by Aspirin. (Fig. 7).

## Discussion

In the current study, the aqueous extract of olive leaves shows no lethality within 24 h. And there were no behavioral changes observed within 2 weeks after the administration of graded doses up to 4000 mg/kg. Our results came in agreement with the result of Clewell et al. (2016), who demonstrated that OLE at doses up to 360, 600, and 1,000 mg/kg/day for 90 days did not induce any mortality and toxicity as recorded by Clewell et al. (2016). Moreover, Guex et al. (2018) showed that oral administration of OLE in groups of rats of both sexes at a single dose of 2,000 mg/kg (acute toxicity) and 100, 200, and 400 mg/kg doses (subacute toxicity) given for 28 days did not cause any toxicity**.** Therefore, according to OECD guidelines for testing of chemicals, OLE is considered safe and approximately falls in category 5 (Co-operation and Development 2008). We concluded that the oral median lethal dose (LD_50_) of OLE was higher than 4000 mg/kg BW and we found that the extract is safe for further work. In addition, we supposed that 100, 200 and 400 mg/kg which equals 1/40th, 1/20th and1/10th of the maximum tested dose of the extract (4000 mg/kg) will be safe in rats. Consequently, these doses were selected for further experiments.

To evaluate the potential anti-inflammatory effect of OLE, we used the carrageenan-induced inflammation assay. As it seems to be the most widely used animal model for evaluating the anti-inflammatory effects of drugs. (Dillingh et al. 2014; Kavimani et al. 2000). It has been evidenced that the acute inflammation caused after carrageenan administration is a biphasic reaction. The initial stage of the edema process combines the actions of histamine, bradykinin, serotonin and substance p, so-called vascular permeability and it lasts for approximately one hour of inflammation, whereas the second delayed stage (after one hour) is mainly a result of lysosomal enzymes, proteases and PG release that mediates the excess secretion of inflammatory fluids at the site of inflammation and formation of edema. This phase is primarily sustained by polymorph nuclear (PMN) cells infiltration into the site of inflammation which triggers the over-secretion of many proinflammatory mediators such as nitric oxide, prostaglandins and cytokines (Wilhelm 1962). In the present study, the aqueous extract of OL exhibited significant (*p* < 0.05) inhibition of paw edema in rats at the doses of 400, 200 and 100 mg/kg, p.o. Furthermore, OLE exerted its anti-inflammatory effect starting from the first measurement of paw thickness, which indicates the ability of the extract to fight against the earliest stages of inflammation as well as the delayed stages. The potent anti-inflammatory activity of OLE continued throughout the measurement period, displaying a marked inhibition percentage at the fifth hour of measurement (46.99% for OLE400). This response tendency of the extract indicates significant peripheral anti-inflammatory properties of the aqueous extract of OLE, which would be related to the presence of polyphenols such as hydroxytyrosol, tyrosol and oleuropein and this may inhibit the formation of leukotriene B4 or LTB4 which has a chemotactic role for leukocytes (macrophages, neutrophils) at sites of inflammation by decreasing the activity of the 5-lipoxygenase (de la Puerta et al. 1999). As reported by Sannigrahi et al. (2011), Flavonoids are known to inhibit the enzyme prostaglandin synthetase, more specifically the endo-peroxidase and are reported to produce an anti-inflammatory effect.

To elucidate the potential anti-inflammatory mechanism of action, we perform further lab analysis on the collected paw tissue to quantify the amount of IL-1, TNF-, COX-2 and NO. The results revealed a significant reduction in all proinflammatory markers that were measured, namely, TNF, IL-1, COX-2 and NO. This reduction was a bit higher than that exerted by the standard drug (diclofenac) and that validates the anti-inflammatory potential of the extract and elucidates the possible mechanism of action by interfering with these inflammatory mediators, and hence attenuates the progression of inflammation. The reduction of the proinflammatory mediators’ level may be related to the inhibition of TNF, IL-1, COX-2 and NO protein expression (Liu et al. 2014). Our results came in the same trend as those reported by (Qabaha et al. 2018) and Fernández-Prior et al. (2021) who reported the in vitro ability of OLE to reduce inflammation through the inhibition of proinflammatory cytokines.

Our findings also revealed that the aqueous extract of OL has the ability to stabilize the rat’s RBCs membrane and protect the lysosomal membrane against damage with a percentage of haemolysis inhibition 25.62, 57.40 and 73.88% at doses of 100, 200 and 400 g/kg, respectively. Such protection and stabilization of a lysosomal membrane can be mediated by preventing the release of a lysosomal enzyme and reducing inflammatory events. Based on the fact that RBCs membrane is similar in structure to the lysosome membrane (Chowdhury et al. 2014). The in vitro ability of OLE to reduce inflammation may be attributed the reduction of proinflammatory cytokines synthesis and release as reported in Teplova et al. (2018). Furthermore, the presence of polyphenols and flavonoids can inhibit the inflammation by targeting the inflammatory NF-jB pathway and inhibiting proinflammatory cytokine-induced chemokine expression (Shen et al. 2017).

## Conclusion

The potent anti-inflammatory activity of olive leaves could be attributed in part to their ability to protect the lysosomal membrane and to reduce the concentration of proinflammatory cytokines at the site of inflammation.

## Data Availability

We confirm that all the data supporting the finding are available in the article and its supplementary data.
